# Association of Non-alcoholic Fatty Liver Disease with Conduction Defects on Electrocardiogram

**DOI:** 10.7759/cureus.1107

**Published:** 2017-03-21

**Authors:** Muhammad A Mangi, Abdul M Minhas, Hiba Rehman, Furquan Pathan, Hong Liang, Sary Beidas

**Affiliations:** 1 GME Internal Medicine, Orange Park Medical Center; 2 GME Internal Medicine, North Florida Regional Medical Center/CyberKnife at North Florida

**Keywords:** nafld, conduction defects, ekg changes

## Abstract

Background: Non-alcoholic fatty liver disease (NAFLD) is a leading cause of liver disease in developed countries. The association of NAFLD with conduction defects is unknown. The aim of our study was to find whether an association exists between conduction defects and NAFLD.

Methods: This is a case-control retrospective study of 700 patients admitted to Orange Park Medical Center, Orange Park, Florida from 2009 to 2015. Patients with a history of alcohol use, congenital heart disease, infiltrative malignancy, and myocarditis were excluded from the study. NAFLD was diagnosed by detection of hepatic steatosis on abdominal ultrasound or computerized tomography (CT) scan. Electrocardiograms (EKGs) were performed on all 700 patients and were interpreted by a cardiologist. Univariate logistic regression was used to assess the association between NAFLD and the variables of demographics, clinical characteristics, medicine use, EKG changes, and conduction defects, while multivariate logistic regression with backward elimination method was performed to determine if NAFLD is one of the most important risk factors for conduction defects.

Results: The study population included 408 patients with NAFLD and 292 patients with No-NAFLD. A total of 155 conduction defects occurred in 140 patients; conduction defects included 25.7% (36) patients with first degree block, 2.1% (three) patients with Mobitz type 1 block, 41.4% (58) patients with right bundle branch block (RBBB), 17.9% (25) patients with left bundle branch block (LBBB), 11.4% (16) patients with bifascicular block, and 12.1% (17) patients with nonspecific intraventricular block. Multivariate logistic regression with backward elimination method identified six risk factors for conduction defects; these included NAFLD (odds ratio (OR) 2.38; 95% confidence interval (CI) 1.51-3.73, p<0.0001), hypertrophy (OR 2.52; 95% CI 1.57-4.05, p=0.0001), congestive heart failure (CHF) (OR 3.05; 95%CI 1.46-6.38, p=0.0031), male sex (OR 1.79; 95%CI 1.19-2.69, p=0.0051), diabetes mellitus (OR 1.63; 95% CI 1.08-2.47, p=0.02), and age (OR 1.04; 95% CI 1.02-1.06, p<0.0001).

Conclusion: NAFLD is associated with conduction defects. Prospective randomized trials are needed to demonstrate that NAFLD causes conduction defects.

## Introduction

Non-alcoholic fatty liver disease (NAFLD) is defined as fatty infiltration of the liver in the absence of alcohol use or infection with viral hepatitis. NAFLD is a common disorder, affecting 25% of the world population and almost 24%-32% of the population in the western world [1]. In the USA, NAFLD affects 30% of the population and is the third most common indication for liver transplant [2-3]. This high prevalence of NAFLD is presumably caused by a high-calorie diet and a sedentary lifestyle [4]. In fact, NAFLD is projected to become the most common cause of end-stage liver disease and liver transplant in the next decade [5-6]. Furthermore, evidence suggests that NAFLD is a multi-system disease that affects the liver and the cardiovascular system leading to structural and functional changes in the heart and the blood vessels. Ultimately these changes are responsible for the increased cardiac morbidity and mortality associated with NAFLD [7-9].

To date, there has been one retrospective study to ascertain the association of NAFLD with conduction defects [10]. The study demonstrated that patients with right bundle branch block (RBBB) are at higher risk of developing NAFLD due to passive congestion of the liver. We extend this line of thought and postulate that patients with NAFLD are at risk for developing other types of conduction defects. In order to test this hypothesis, we conducted a large population-based case-control study of adult men and women at Orange Park Medical Center (OPMC) in Florida.

## Materials and methods

The study protocol was approved by the OPMC Graduate Medical Education Research committee as a minimal risk study because the study is a retrospective chart review with no patient contact.

### Study design

**Figure 1:**
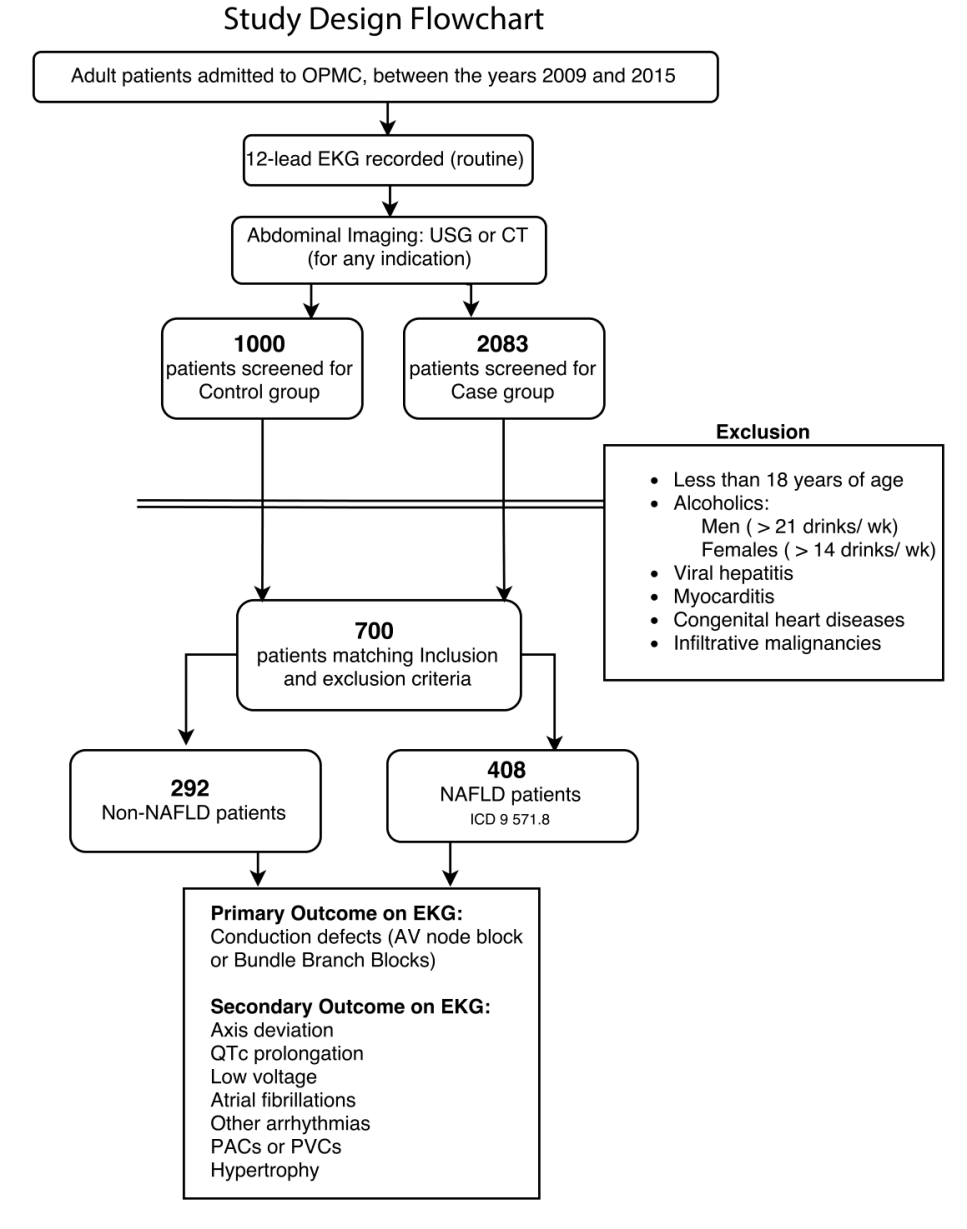
Study design flowchart

This was a retrospective case-control study designed to test the association between NAFLD and conduction defects (Figure 1). NAFLD was an incidental finding in patients admitted for other reasons. NAFLD was diagnosed by detection of hepatic steatosis on abdominal ultrasonography (USG) or computerized tomography (CT) scans. Patients were divided into two groups: group-1 included patients who had NAFLD diagnosed on abdominal imaging and group-2 were controls who did not have NAFLD on abdominal imaging. Controls (No-NAFLD) were randomly selected from the general population of hospitalized adult patients, who also had an electrocardiogram (EKG) and abdominal imaging studies performed during hospitalization. All EKGs were interpreted by a cardiologist independent of the study.

### Study population

The study population included adult patients (>18 years of age) admitted to the OPMC from 2009 to 2015. All patients had abdominal imaging and EKG studies ordered. Based on abdominal imaging, the population was divided into NAFLD versus No-NAFLD cases. Patients were excluded if there was a history of alcohol use, defined as >21 drinks/week for men and >14 drinks/week for women. Other exclusions included acute viral hepatitis, chronic viral hepatitis, congenital heart disease, infiltrative malignancy, myocarditis or cardiac surgeries. Also, patients who did not have an abdominal USG, CT scan abdomen or EKG were excluded from the study.

### Predictor variables

Baseline demographic characteristics collected included age, gender, race, obese, chronic conditions (asthma/chronic obstructive pulmonary disease (COPD), congestive heart failure (CHF), diabetes mellitus, hypertension) ischemic heart disease, NAFLD, medication use (beta-blockers, calcium channel blockers, digoxin, amiodarone, adenosine), cirrhosis, thyroid disorders, smoking, cocaine use (Table 1). We did not include body mass index (BMI) in our study due to lack of availability of height in the medical record for most patients.

### Outcome variables

Conduction defect identified on EKG was the primary outcome variable. The EKG changes for conduction defects were determined by reviewing EKG tracings already verified by a cardiologist. Secondary outcomes for this study included the presence of other EKG changes (for example, atrial fibrillation, premature atrial contractions (PACs), premature ventricular contractions (PVCs), axis deviation, low voltage, prolonged QTc interval, hypertrophy, and ST wave changes).

### Statistical analysis

Univariate logistic regression analysis was used to assess the association between NAFLD, baseline demographics, clinical characteristics, medicine use, EKG changes, and conduction defects (Table 2). Multivariate logistic regression analysis with backward elimination method was performed to determine if NAFLD is a risk factor for conduction defects from 19 selected predictors/factors (age, sex, race, obesity, coronary artery disease (CAD), hypertension, diabetes mellitus, CHF, smoking, COPD, asthma, thyroid disorder, antipsychotic medicine, hyperlipidemia, and NAFLD) (Table 3). The risk estimates were reported as odds ratios (OR) with 95% confidence intervals (CI). A p-value < 0.05 was considered statistically significant. R version 3.3.1 (University of Auckland, New Zealand) was primarily used for statistical analysis and SAS 9.4 (SAS Institute Inc., Cary, NC) was used to validate R output.

## Results

A total of 700 patients were included in the study. NAFLD was identified on abdominal imaging in 408 patients. In the No-NAFLD group, there were 292 patients. The median age was 58 years (standard deviation (SD)=15.3 years). There were 293 males (41.9%), and 407 females (58.1%). Most patients were Caucasian (n=534, 76.3%). Other baseline characteristics are listed below (Table 1). A total of 155 conduction defects were identified in 140 patients demonstrated on EKG (Table 4).

**Table 1:** Demographics and clinical characteristics of the study population

	ALL		NAFLD		No NAFLD	
	N,% or Median, SD		N,% or Median, SD		N,% or Median, SD	
Age (median)(SD)	57.9	(15.3)	59	(13.6)	56.3	(17.5)
Sex (males)	293	(41.8%)	178	(43.6%)	115	(39.4%)
(females)	307	(58.2%)	230	(56.4%)	177	(60.8%)
Race						
Caucasian	534	(76.4%)	318	(80%)	216	(74.2%)
African American	98	(14%)	45	(11%)	53	(18.3%)
Hispanic	17	(0.02%)	16	(3.9%)	1	(0.3%)
Other	50	(0.07%)	29	(7.1%)	21	(7.2%)
Smokers	237	(33.8%)	142	(34.8%)	96	(32.9%)
Illicit Drug Users	73	(10.4%)	46	(11.3%)	27	(9.3%)
Hypertensive	421	(60.1%)	262	(64.2%)	159	(54.5%)
CAD	94	(13.4%)	45	(11.0%)	49	(16.8%)
Hyperlipidemia	230	(32.8%)	123	(30.2%)	107	(36.6%)
Diabetics	224	(32%)	156	(38.2%)	68	(23.3%)
Obese	339	(48.4%)	214	(52.5%)	125	(42.8%)
CHF	38	(0.05%)	23	(5.6%)	15	(5.1%)

### Univariate analysis

Univariate analysis of EKG findings, (conduction defect, PACs/PVCs, hypertrophy, axis deviation, ST wave changes, low voltage, and QTc prolongation) identified a positive association with NAFLD (p-value <0.05). Atrial fibrillation showed no association with NAFLD (Table 2).

The proportion of patients with a conduction defect on EKG was higher in patients with NAFLD (107/408, 26.2%) compared to the No-NAFLD group (33/292, 11.3%), (p-value <0.001). When conduction defects were further stratified (Table 4) into AV block and bundle branch block, AV block was not significantly associated with NAFLD (6.9% in NAFLD vs 3.8% No-NAFLD (p-value 0.0828). However, bundle branch block was significantly associated with NAFLD (22.1% in NAFLD vs 8.9% in No-NAFLD (p-value <0.001).

**Table 2:** Univariate analysis of demographics, clinical characteristics, medicine, and EKG changes by NAFLD TCA - tricyclic antidepressant, SSRI - selective serotonin reuptake inhibitors, CCB - calcium channel blocker.

Variables	NAFLD (408)		No-NAFLD (292)		Odds ratio (95% CI)	p-value*
	N,% or Mean, SD		N,% or Mean, SD			
Mean age, year (SD)	59.0	(13.6)	56.3	(17.5)	1.01 (1.00-1.02)	0.0203
Sex (male)	178	(43.6)	115	(39.4)	1.19 (0.88-1.62)	0.2619
Race (African American)	45	(11.0)	53	(18.2)	0.56 (0.36-0.86)	0.0080
Smokers (current/former)	142	(34.8)	96	(32.9)	1.09 (0.79-1.50)	0.5956
Hypertensive	262	(64.2)	159	(54.5)	1.50 (1.11-2.04)	0.0094
CAD	45	(11.0)	49	(16.8)	0.62 (0.40-0.95)	0.0288
Hyperlipidemia	123	(30.2)	107	(36.6)	0.75 (0.54-1.03)	0.0715
Diabetics Mellitus	156	(38.2)	68	(23.3)	2.04 (1.46-2.86)	<0.0001
Obese	214	(52.5)	125	(42.8)	1.47 (1.09-1.99)	0.0120
CHF	23	(5.6)	15	(5.1)	1.11 (0.57-2.16)	0.7676
Cirrhosis	73	(17.9)	2	(0.68)	31.59 (7.69-129.84)	<0.0001
COPD	59	(14.5)	34	(11.6)	1.28 (0.82-2.02)	0.2798
Asthma	12	(2.9)	14	(4.8)	0.60 (0.27-1.32)	0.2054
PAC/PVC	86	(21.1)	29	(9.9)	2.42 (1.54-3.80)	0.0337
Thyroid disorder	78	(19.1)	41	(14.0)	1.45 (0.96-2.19)	0.0789
Illicit Drug Users	46	(11.3)	27	(9.3)	1.25 (0.76-2.06)	0.3874
Beta Blocker	130	(31.9)	92	(31.5)	1.02 (0.74-1.40)	0.9206
Digoxin	4	(1.0)	1	(0.3)	2.88 (0.32-25.91)	0.3450
TCA	13	(3.2)	5	(1.7)	1.89 (0.67-5.36)	0.2321
SSRI	75	(18.4)	37	(12.7)	1.55 (1.01-2.38)	0.0432
CCB	80	(19.6)	59	(20.2)	0.96 (0.66-1.40)	0.8448
Antipsychotic	21	(5.2)	14	(4.8)	1.08 (0.54-2.16)	0.8339
Amiodarone	0	(0.0)	5	(1.7)	<0.001 (<0.001 - >999.9)	0.9797
Adenosine	0	(0.0)	0	(0.0)	NA	NA
Atrial fibrillation	45	(11.0)	33	(11.3)	0.97 (0.60-1.57)	0.9101
Low Voltage	57	14.0)	17	(5.8)	2.63 (1.49-4.62)	0.0008
QT-prolongation	93	(22.8)	16	(5.5)	5.09 (2.92-8.86)	<0.0001
ST-change	195	(47.8)	115	(39.4)	1.41 (1.04-1.91)	0.0274
Anterioseptal-ST	49	(12.0)	33	(11.3)	1.07 (0.67-1.71)	0.7749
Anteriolateral-ST	45	(11.0)	30	(10.3)	1.08 (0.66-1.76)	0.7514
Interior-ST	33	(8.1)	20	(6.9)	1.20 (0.67-2.13)	0.5417
Posterior-ST	3	(0.7)	0	(0.0)	>999 (<0.01 - >999.9)	0.9845
Nonspecific-ST	124	30.4)	50	(17.1)	2.11 (1.45-3.06)	<0.0001
Axis deviation	91	(22.3)	24	(8.2)	3.21 (1.99-5.17)	<0.0001
Hypertrophy	91	(22.3)	33	(11.3)	2.25 (1.46-3.47)	0.0002
Conduction Defect	107	(26.2)	33	(11.3)	2.79 (1.83-4.26)	<0.0001
AV block	28	(6.9)	11	(3.8)	1.88 (0.92-3.84)	0.0828
Bundle Branch Block	90	(22.1)	26	(8.9)	2.90 (1.82-4.61)	<0.0001

### Multivariate analysis

Multivariate logistic regression with backward elimination identified six risk factors for conduction defects including; NAFLD (OR 2.38; 95% CI 1.51-3.73, p <0.0001), hypertrophy (OR 2.52; 95% CI 1.57-4.05, p=0.0001), CHF (OR 3.05; 95% CI 1.46-6.38, p=0.0031), male sex (OR 1.79; 95% CI 1.19-2.69, p=0.0051), diabetes mellitus (OR 1.63; 95% CI 1.08-2.47, p=0.02), and age (OR 1.04; 95% CI 1.02-1.06, p<0.0001) (Table 3).

**Table 3:** Multivariate logistic regression analysis for conduction defect using backward elimination method

Variables#	Conduction Defect (140)		No-Conduction Defect (560)		Odds ratio (95%CI)	p-value
	N,% or Mean, SD		N,% or Mean, SD			
Mean age, year (SD)	64.5	(14.3)	56.3	(15.3)	1.04 (1.02-1.06)	<0.0001
Sex (male)	71	(50.7)	222	(39.6)	1.79 (1.19-2.69)	0.0051
Diabetics Mellitus	62	(44.3)	162	(28.9)	1.63 (1.08-2.47)	0.0205
CHF	17	(12.1)	21	(3.8)	3.05 (1.46-6.38)	0.0031
Hypertrophy	42	(30.0)	82	(14.6)	2.52 (1.57-4.05)	0.0001
NAFLD	107	(76.4)	301	(53.8)	2.38 (1.51-3.73)	< 0.0001

**Table 4:** Conduction defect distribution

Variables	No-NAFLD (33)		NAFLD (107)		Total (140)	
	n	%	n	%	n	%
1^st^ degree AV block	11	7.1	25	16.1	36	23.2
Mobitz type 1	0	0.0	3	2.1	3	2.1
Right bundle branch block	14	9.0	44	28.4	58	37.4
Left bundle branch block	4	2.6	21	13.5	25	16.1
Bi-fascicular block	1	0.6	15	9.7	16	10.3
Nonspecific intraventricular block	7	4.5	10	6.5	17	11.0
Total conduction defects	37	23.9	118	76.1	155	100.0

## Discussion

The major finding of this study is that patients diagnosed with NAFLD showed a significant association with conduction defects. This association was independent of numerous other risk factors after adjusting for confounders.

Prior studies have shown associations between NAFLD and CAD [11-13]. However, few studies have evaluated the association of NAFLD with electrical abnormalities of the heart. To our knowledge, this is the first study to evaluate for the presence of conduction defects and describe EKG abnormalities found in patients with NAFLD.

The underlying pathophysiological mechanism responsible for the association between NAFLD and conduction defect is not well understood. NALFD might be a marker for ectopic fat deposition in myocardium and pericardium that promotes cardiovascular disease. A recent study has shown an association between intra-hepatic and myocardial triacylglycerol content and that increased pericardial fat is associated with increased prevalence of atrial fibrillation [14-15]. Further, adipocytes in retrosternal, epicardial tissue have been shown to exert effects on ion currents in rabbit left atria, leading to arrhythmias [16]. NAFLD may potentially contribute to the development of cardiovascular complications as demonstrated by the increased production of pro-inflammatory cytokines such as C-reactive protein, interleukin-6, tumor necrosis factor – alpha and prothrombotic factors [17-18]. These markers have been associated with structural changes in the heart and a higher rate of arrhythmias [19-21].

This study has several limitations. First, NAFLD was diagnosed by USG or CT scan, instead of tissue biopsy, the gold standard. Studies have shown that USG has a sensitivity of 60% to 94% and specificity of 84% to 95%, while CT scan has a sensitivity of 82% and specificity of 100%; so neither USG nor CT scan is able to reliably detect hepatic lipid content that is less than 30% [22-24]. Hence, we probably missed mild to moderate cases of NAFLD [24]. Furthermore, we were unable to differentiate NAFLD from non-alcoholic steatohepatitis, as no tissue biopsy results were available. Second, patients were not primarily admitted for NAFLD, rather NAFLD was an incidental finding identified using International Classification of Diseases, Ninth Revision (ICD-9) codes. Finally, we cannot exclude the effect of unmeasured or unknown confounding factors. For instance, although NAFLD is strongly associated with obesity, we were unable to exclude the confounding effect of BMI on conduction defects due to unavailability of uniformly identifying patient height.

Several studies have demonstrated increased cardiovascular morbidity and mortality in NAFLD patients [25-26]. It is plausible that healthcare providers should treat patients with NAFLD for various cardiovascular risk factors in order to reduce their cardiovascular morbidity and mortality. Lifestyle modification, including weight loss, dietary modifications, and physical activity are the first line treatment for NAFLD and were shown to improve outcomes in patients with NAFLD [27-28]. More recently, thiazolidinediones (pioglitazone and rosiglitazone) and liraglutide were recommended for the management of NAFLD [29-30].

## Conclusions

The role of NAFLD as a novel risk factor for cardiac disease has been extensively researched in the last decade. Previous studies have shown an association between NAFLD and structural and metabolic cardiac changes. Still, the data is relatively scarce for the association between NAFLD and arrhythmogenic cardiac abnormalities. Our study suggests that NAFLD is associated with conduction defects. Larger studies are needed to find out the causal relationship as well as the pathophysiological pathways that connect NAFLD and arrhythmogenic abnormalities of the heart.
